# Complete Genome Analysis of Three *Acinetobacter baumannii* Clinical Isolates in China for Insight into the Diversification of Drug Resistance Elements

**DOI:** 10.1371/journal.pone.0066584

**Published:** 2013-06-24

**Authors:** Lingxiang Zhu, Zhongqiang Yan, Zhaojun Zhang, Qiming Zhou, Jinchun Zhou, Edward K. Wakeland, Xiangdong Fang, Zhenyu Xuan, Dingxia Shen, Quan-Zhen Li

**Affiliations:** 1 Department of Immunology and Internal Medicine, The University of Texas Southwestern Medical Center, Dallas, Texas, United States of America; 2 CAS Key Laboratory of Genome Sciences and Information, Beijing Institute of Genomics, Chinese Academy of Sciences, Beijing, China; 3 Department of Clinical Microbiology, General Hospital of People’s Liberation Army, Beijing, China; 4 State Key Laboratory of Mycology, Chinese Academy of Sciences, Beijing, China; 5 Department of Molecular and Cell Biology and Center for Systems Biology, The University of Texas at Dallas, Richardson, Texas, United States of America; Charité-University Medicine Berlin, Germany

## Abstract

**Background:**

The emergence and rapid spreading of multidrug-resistant *Acinetobacter baumannii* strains has become a major health threat worldwide. To better understand the genetic recombination related with the acquisition of drug-resistant elements during bacterial infection, we performed complete genome analysis on three newly isolated multidrug-resistant *A. baumannii* strains from Beijing using next-generation sequencing technology.

**Methodologies/Principal Findings:**

Whole genome comparison revealed that all 3 strains share some common drug resistant elements including carbapenem-resistant *bla*_OXA-23_ and tetracycline (*tet*) resistance islands, but the genome structures are diversified among strains. Various genomic islands intersperse on the genome with transposons and insertions, reflecting the recombination flexibility during the acquisition of the resistant elements. The blood-isolated BJAB07104 and ascites-isolated BJAB0868 exhibit high similarity on their genome structure with most of the global clone II strains, suggesting these two strains belong to the dominant outbreak strains prevalent worldwide. A large resistance island (RI) of about 121-kb, carrying a cluster of resistance-related genes, was inserted into the *ATPase* gene on BJAB07104 and BJAB0868 genomes. A 78-kb insertion element carrying *tra*-locus and *bla*_OXA-23_ island, can be either inserted into one of the *tniB* gene in the 121-kb RI on the chromosome, or transformed to conjugative plasmid in the two BJAB strains. The third strains of this study, BJAB0715, which was isolated from spinal fluid, exhibit much more divergence compared with above two strains. It harbors multiple drug-resistance elements including a truncated AbaR-22-like RI on its genome. One of the unique features of this strain is that it carries both *bla*_OXA-23_ and *bla*_OXA-58_ genes on its genome. Besides, an *Acinetobacter lwoffii* adeABC efflux element was found inserted into the ATPase position in BJAB0715.

**Conclusions:**

Our comparative analysis on currently completed *Acinetobacter baumannii* genomes revealed extensive and dynamic genome organizations, which may facilitate the bacteria to acquire drug-resistance elements into their genomes.

## Introduction

*Acinetobacter baumannii* is an important opportunistic pathogen of hospital acquired infection, particularly in intensive care units, which is usually responsible for up to 10% of hospital-acquired infections and increases mortality up to 70% [1]–[4]. *A. baumannii* often causes outbreaks of infection and can survive for long periods in the hospital environment [5]. Moreover, *A. baumannii* shows a strong ability to acquire foreign DNA such as drug resistance and pathogenicity, which makes it to acquire genetic diversity and overcomes the antibiotic selection pressure [6]. The antimicrobial resistance in this nosocomial pathogen is mainly caused by inactivating enzymes such as β-lactamases, alteration of membrane porin channels, and mutations that change cellular functions.

Recently, increasing resistance to carbapenems in *A. baumannii* has emerged which severely limits the treatment options for this pathogen. The most important resistance mechanism is mediated by producing class D β-lactamases with carbapenemase activity, such as *bla*_OXA-23_-like, *bla*_OXA-24_-like, *bla*_OXA-40_-like, and *bla*_OXA-58_-like genes in *A. baumannii* [7]–[10]. Among them the *bla*_OXA-23_ gene, first identified in Scotland, has been found worldwide spread [11]–[20].

Next generation sequencing (NGS) technology provides an ability to evaluate resistance mechanisms, pathogenicity and evolution of bacterial pathogens on genome-wide level and has been proved to be useful to thoroughly understand the basic features of pathogens in order to ultimately control the spread of pathogen infections and to develop effective treatments. The whole genomes of many clinical important and prevalent *A. baumannii* representatives have been sequenced [21]–[32]. The identification of the genomic components of *A. baumannii* provides a scaffold to rapidly evaluate the genomic organization and epidemiological information of novel clinical *A. baumannii* isolates.

We reported here the genome sequences of three recently isolated multidrug-resistant (MDR) strains from Beijing, China (BJAB strains), including BJAB07104, BJAB0868, and BJAB0715, which were isolated from different clinical samples but all have *bla*_OXA-23_ gene. Genome comparison analysis was performed to determine how the differences of genomic organization and sequence divergence are related to the observed resistance and pathogenesis phenotypes.

## Results and Discussion

### Susceptibility Profiles and Multilocus Sequence Typing (MLST)

Three representative MDR *A. baumannii* strains, BJAB0715, BJAB0868 and BJAB07104, which were isolated from different clinical samples in Beijing during March 2007 and April 2008, were selected for whole-genome sequencing. The three strains were isolated from bloodstream (BJAB07104), ascites (BJAB0868) and cerebrospinal fluid (BJAB0715), respectively, and showed a similar susceptibility pattern. All of them are resistant to almost all currently available antibiotics including imipenem, amikacin, minocycline, ciprofloxacin, levofloxacin, piperacillin, piperacillin/tazobactam, ceftazidime, cefotaxime, cefepime, cefoperazone/sulbactam (1∶1) and meropenem; but susceptible to polymyxin B. The drug-susceptibility profiles were showed in Table 1.

**Table 1:** Susceptibility profiles of three MDR strains.

Antibiotics	BJAB0715		BJAB0868		BJAB07104	
	MIC(mg/L)	R/S	MIC(mg/L)	R/S	MIC(mg/L)	R/S
amikacin	256	R	>256	R	>256	R
caftazidime	16	R	128	R	128	R
cefepime	32	R	256	R	128	R
cefotaxime	64	R	>256	R	>256	R
ciprofloxacin	16	R	64	R	32	R
imipenem	64	R	128	R	>64	R
levofloxacin	8	R	16	R	8	R
meropenem	64	R	64	R	64	R
minocycline	64	R	8	S	16	R
piperacillin	>512	R	>256	R	>512	R
tazobactam	>128	R	>256	R	>128	R
polymyxin	2	S	2	S	2	S
Tetracyclines	>16	R	>16	R	>16	R

MLST was first performed for investigating the population structure of three *A. baumannii* clinical isolates [33]. An *A. baumannii* database (www.pasteur.fr/recherche/genopole/PF8/mlst/Abaumannii.html) was used to analyze sequences of the 7 housekeeping genes (*cpn60, fusA, gltA, pyrG, recA, rpIB and rpoB)*. We found that the isolates of BJAB07104 and BJAB0868 show the same allelic profile (cpn60-2, fusA-2, gltA-2, pyrG-2, recA-2, rpIB-2 and rpoB-2), which corresponds to European clone II (also called global clone II (GC II)), and were recommended to be designated by ST2 or CC2 (where CC stands for clonal complex) for uniform nomenclature. BJAB0715 strain shows a different allelic profile (cpn60-1, fusA-3, gltA-10, pyrG-1, recA-4, rpIB-4 and rpoB-4), and was recommended to be designated by ST23 or CC10.

### Whole Genome Sequencing of the Three*A. baumannii* Strains

**Figure 1:**
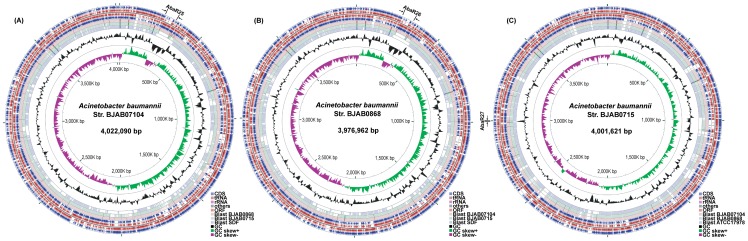
Circular representation of genomes of three*Acinetobacter baumannii* strains. (a) BJAB07104. (b) BJAB0868. (c) BJAB0715. Circles display (from outside in order of) (i) coding regions in the clockwise direction; (ii-iii) open reading frames (>100 codons) in the clockwise and counterclockwise direction respectively; (iv) coding regions in the counterclockwise direction; (v-vi) comparison with three selected genomes by BLAST (BJAB0868, BJAB0715 and SDF for BJAB07104, BJAB07104, BJAB0715 and SDF for BJAB0868, BJAB07104, BJAB0868 and ATCC17978 for BJAB0715); (vii) GC content; and (viii) G-C skew. The plot was produced by CGView server (http://stothard.afns.ualberta.ca/cgview_server/index.html) [75]. The locus of AbaR-like resistance islands were marked beside the circular genomes.

Pair-end sequencing produced >9 million 75-bp nucleotide reads for each of the three strains. After *de novo* assembly and manual gap-closing by PCR and re-sequencing using Sanger sequencing method, the complete genomes of BJAB07104, BJAB0868 and BJAB0715 strains yield 4,022,090-bp, 3,976,962-bp, and 4,001,621-bp with a G+C content of 38.96%, 38.93 and 38.87% respectively (Fig. 1a, 1b and 1c). The characteristics of the three genomes are listed in Table 2. Using the NCBI Prokaryotic Genome Automatic Annotation Pipeline (PGAAP) and the genome of ACICU strain (CP000863.1) as a reference sequence, we predicted 3,869, 3,816, and 3,850 potential protein-coding genes from BJAB07104, BJAB0868, and BJAB0715 genomes respectively. Among them, 1,374 (35.51%), 1,325 (34.72%), and 1,360 (35.32%) genes in these three genomes respectively encode hypothetical proteins (Table 2). The number of genes is comparable with other previously sequenced *A. baumannii* strains [21]–[32].

**Table 2:** General features of*A. baumannii* BJAB07104, BJAB0868 and BJAB0715 genomes.

Characteristic	BJAB07104	BJAB0868	BJAB0715	pBJAB07104	p1BJAB0868	p2BJAB0868	pBJAB0715
GenBank Accession No.							
Main genome size	4022090	3976962	4001621	20139	8721	20139	52268
No. of plasmid	1	2	1		/		/
Whole genome size	4042229	4005822	4053889	20139	8721	20139	52268
G+C content (%)	38.96	38.93	38.87	47.07	34.34	47.09	40.43
No. of genes	3933	3881	3926	20	10	20	60
No. of protein-encoding genes	3869	3816	3850	20	10	20	60
No. of predicted genes	1374	1325	1360	8	7	7	34
No. of tRNAs	74	75	73		/		/
No. of rRNAs	18	18	18		/		/
No. of insertion sequences (ISAba1)	21 (17)	19 (13)	26 (14)	3 (0)	0	3 (0)	4 (0)

**Figure 2:**
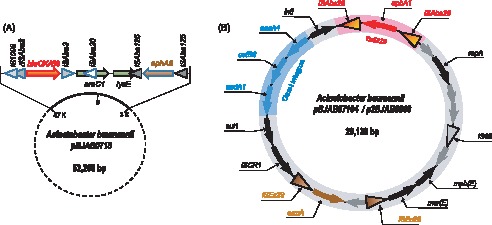
The structures of representative plasmids containing drug resistance genes. (a) pBJAB0715 contains *bla*_OXA-58_ flanked by two ISAba3 elements, and *aphA6* flanked by two ISAba125 elements. (b) pBJAB07104 and p2BJAB0868 containing *aphA1* in transposon Tn6210 (in red) and genes of class I integron (in blue).

Sequencing analysis also identified four plasmids from these three BJAB strains, of which two are from BJAB0868, one from BJAB07104, and one from BJAB0715 (Table 2). The BJAB0715 strain harbors a 52,268-bp plasmid (pBJAB0715) with little similarity to any published plasmids in NCBI database. It contains 60 protein coding genes including four antibiotic resistance genes *bla*_OXA-58_, *aac3′-1*, *aphA6* and *cm1A1* (Fig. 2a). For the two plasmids in BJAB0868 strain, p1BJAB0868 is 8,721-bp containing 10 protein coding genes and almost identical with the published plasmid pAB0057 (99.8%) [22]; p2BJAB0868 is 20,139-bp and near-perfectly identical with the plasmid pBJAB07104 from BJAB07104 strain, of which both share high similarity with the published plasmid pZJ06 (92%) [27]. Each of p2BJAB0868 and pBJAB07104 carries 20 protein-coding genes including six drug resistance genes on class I integron and *aphA1* transposon (Fig. 2b). Except the plasmid p1BJAB0868 which was estimated having 15 copies in a cell, each of the rest three plasmids only have one copy based on the average sequencing coverage depth of NGS data. The genome sequences of the three *A. baumannii* strains and the four plasmids have been deposited into GenBank with accession numbers CP003846 (BJAB07104), CP003887 (pBJAB07104), CP003849 (BJAB0868), CP003850 (p1BJAB0868), CP003888 (p2BJAB0868), CP003847 (BJAB0715), and CP003848 (pBJAB0715).

### Phylogenetic Analysis of*A. baumannii* Genomes

**Figure 3:**
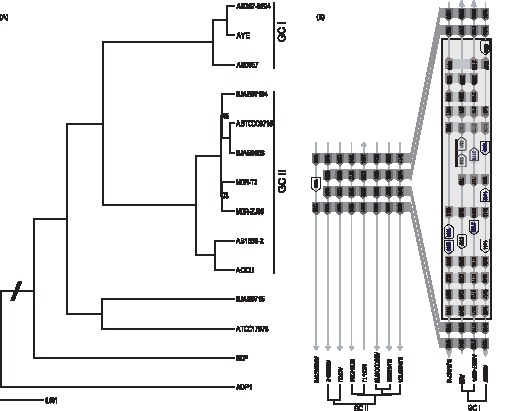
Sequence comparison of BJAB strains with other*A. baumannii* strains. (a) Phylogenetic analysis of 13 *A. baumannii* strains with *Acinetobacter baylyi* ADP1 as outgroup. 1,119 orthologous proteins identified from 14 genomes were aligned by CLUSTALW and PHYLIP software package was used to construct the tree. 1000 replicates were used in bootstrap analysis. (b) An insert region in the genome of BJAB0715, was found in all three genomes of GC I strains but not in GC II strains.

Whole genome phylogenetic analysis was performed by using the conserved proteins among the three BJAB strains and ten other *A. baumannii* strains with complete genomes in GenBank. These include seven MDR strains (AYE, AB0057, ACICU, AB16562, ABTCDC0715, MDR-TJ, and MDR-ZJ06), two susceptible strains (ATCC17978 and AB307-0294) and a non-clinical strain SDF isolated from a human body louse. ADP1, a soil-living bacterium *A. baylyi* strain was used as outgroup for comparison. All clinical isolated *A. baumannii* strains contain a genetically highly homogeneous core genome which encodes proteins with functions involved in DNA replication, transcription, and translation, as well as many metabolic pathways. By using reciprocal best BLAST matches, we identified 1,119 conserved orthologous proteins among all 14 *Acinetobacter* isolates including *A. baylyi* ADP1 (Table S1). The number of conserved orthologous proteins increases to 1,331 among the 13 *A. baumannii* strains (exclude *A. baylyi* ADP1), and 3,115 among the three newly sequenced BJAB strains. The phylogenetic pattern within *A. baumannii* was investigated by neighbor-joining analysis of these 1,119 orthologous protein sequences with ADP1 as outgroup (Fig. 3a). Based on the phylogenetic data, the three strains (AYE, AB307-0294 and AB0057) which belong to global clone I (GC I) were grouped together. Two of the three BJAB strains (BJAB07104 and BJAB0868), along with 4 previously reported Asia strains, including MDR-ZJ06 (China), MDR-TJ (China), ABTCD0715 (Taiwan) and AB1656-2 (Korean), were grouped together with ACICU, a strain of global clone II (GC II) group. Interestingly, BJAB0715 is separated with all of the MDR strains (Fig. 3a), which may suggest BJAB0715 has a different origin comparing with other drug-resistant strains.

### Resistance Island (RI) Containing*bla*_OXA-23_ in Different *A. baumannii* Strains

**Figure 4:**
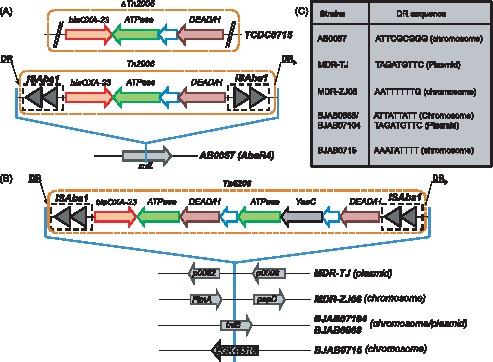
Transposons containing drug resistance gene*bla*_OXA-23_ in different *A. baumannii* strains (a) Truncated Tn2006 in TCDC0715 and complete Tn2006 in AbaR4 reported in AB0057 strain; (b) Tn6206 found in three BJAB strains and Tn2009 in MDR-ZJ06 and MDR-TJ strains. (c) 9-bp target site direct repeat sequences (DR sequences) of ISAba1 elements (in bold font).

Resistance islands (RIs) are large insertions containing a collection of horizontally transferred genes related to antibiotic inactivation and efflux. The RIs can be carried on bacterial chromosome or on plasmid, and antibiotic resistance genes are usually interspersed with mobile genetic elements such as IS and transposons [34]. *bla*_OXA-23_ containing RI was identified in the genome of all three BJAB strains. The *bla*_OXA-23_ gene is associated with carbapenems resistance and has been identified in clinical *A. baumannii* isolates around the world [11]–[20]. But the structure and genome location of *bla*_OXA-23_ containing RI is different among strains. In AB0057 and TCDC0715, the *bla*_OXA-23_ is carried by transposon Tn2006 (or a truncated form) in AbaR4 and inserted into the *sulf* gene region in AB0057 strain (Fig. 4a) [22], [30]. However, in the three BJAB strains, the *bla*_OXA-23_ resistance islands are different from AbaR4 by lack of *uspA* and *sup* genes but containing *yeeB* gene. These islands in three BJAB strains have the same structure as those in pABTJ1 and MDR-ZJ06 [27], [31], but the insertion positions are different. In MDR-ZJ06 and pABTJ1 (MDR-TJ), the *bla*_OXA-23_ is located in an 8,423-bp transposon (Tn2009) and inserted into either the pilus assembling gene cluster (Fig. 4b) on chromosome (MDR-ZJ06) or present on a plasmid (MDR-TJ). In the three BJAB strains, the *bla*_OXA-23_ are all located in an 8,426-bp transposon (designated as Tn6206) which has high sequence similarity (99.9%) with Tn2009 [27]. Tn6206 carries 8 protein coding genes including *bla*_OXA-23,_ two copies of *ATPase* and *DEAD/H*, *YeeB, YeeC* and a few hypothetical proteins. There are two copies of the same-direction ISAba1 elements at both sides of the transposon and a 16-bp inverted repeat [5′-CTCTGTACACGA(T/C)AAA-3′] flanking the ISAba1 elements. In BJAB0715, the Tn6206 is inserted into the *EGK48316* gene on chromosome and a 9-bp target site direct repeat sequence (5′-AAATATTTT-3′) was identified on both side of the insertion sequence (Fig. 4b and 4c). However, in BJAB07104 and BJAB0868, Tn6206 is inserted into the chromosome inside of *tniB* gene and interrupts it. Furthermore, we observed that a 78-kb insertion element containing Tn6206 and *tra*-locus in BJAB0868 and BJAB07104 strains could be either site-specifically integrated into chromosome, or excised as a circular plasmid which was confirmed by PCR amplification and Southern blot hybridization (Fig. S1–S2). Three types of plasmid could be formed from this 78-kb insertion sequence, the *tra*-locus alone, Tn6206 alone or *tra*-Tn6206 conjugation, indicating that the *tra*-locus and Tn6206 can transfer freely between chromosome and plasmid. When integrated into the chromosome, *tra*-locus and Tn6206 can be in two different orders (5′-Tn6206-*tra*-locus-3′, or 5′-*tra*-locus-Tn6206-3′), indicating that the plasmid containing *tra*-locus and Tn6206 is integrated into the chromosome by homologous recombination at different ISAba1 sites of this plasmid to form a 121-kb RI in BJAB07104 and BJAB0868. The 9-bp target site direct repeat sequences (DR) were found at both sides of the inserted sequences, but the DR sequences are different when this insertion is integrated in chromosome (ATTATTATT) or on plasmid (TAGATGTTC). Our data suggested that HGT mediated by plasmids is a key contributor for evolution of the clinical *A. baumannii* strains by vectoring ecologically important traits between strains and species. The transfer of the mobile genetic element between chromosome and plasmid may facilitate the rapid spreading of the resistant genes among *A. baumannii* strains [35].

### Novel AbaR-like Resistance Islands

**Figure 5:**
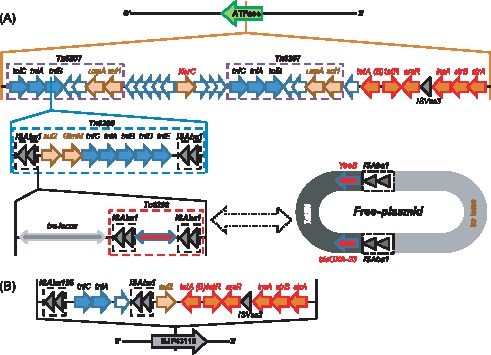
Structure of resistance islands AbaR25-27 containing drug resistant gene*tetA*. (a) AbaR25 in BJAB07104 and AbaR26 in BJAB0868 are both inserted into the *ATPase* gene. Transposons Tn6206-6209 are showed in dashed rectangles. While Tn6208 (in BJAB07104) is flanked by two ISAba1 elements, Tn6209 (in BJAB0868) only has the left flanking ISAba1 element. When being present in chromosome, Tn6206 and *tra* system are inserted into the gene *tniB*, while this region can also form free plasmids. (b) AbaR27 in BJAB0715 is inserted into gene *EJP43116*, which produces a protein 100% identical to a hypothetical protein found in *A. baumannii* OIFC032 strain.

The AbaR-like structure containing clusters of drug resistance genes has been reported in many drug-resistant *A. baumannii* strains [21]–[32], [36]. The largest AbaR-like RI in *A. baumannii* reported by far was the 86-kb AbaR1 in AYE, which harbored a cluster of 45 resistance-related genes [32]. In this study, we identified 3 novel AbaR-like resistance islands, designated as AbaR25, AbaR26 and AbaR27, from BJAB07104, BJAB0868 and BJAB0715, respectively (Fig. 5). The AbaR25 in BJAB07104 is about 121.7-kb containing 141 protein-coding genes including 7 antibiotic resistance genes (*sul1, tetA(B), arsR, strB, strA, bla_OXA-23_* and *sul2*). AbaR25 is inserted into the *ATPase* (*comM*) gene position on chromosome with the identical 5-bp direct repeat (5′-accgc-3′) flanking both ends of the insertion sequence. The AbaR26 in BJAB0868 is almost identical to AbaR25 except that the 1,180-bp ISAba1 element on the right side of transposon Tn6208 is deleted in AbaR26 (Fig. 5a).

Sequence analysis revealed that the backbone of the AbaR25/AbaR26 is a 34-kb insertion island which has similar structure as the AbaR22 in MDR-ZJ06 [27]. This 34-kb backbone consists of two copies of Tn6207, a *tet* island containing the tetracycline efflux pump and its regulator genes, *tetA(B)* and *tetR*, and a truncated Tn5393-like island containing aminoglycoside resistance genes *strB* and *strA* (Fig. 5a). An 87-kb fragment containing the *tra*-locus, Tn6206 and Tn6208 (Tn6209) are inserted into the *tniB* gene position in one of the Tn6207 locus in the backbone (Fig. 5a). This insertion sequence contains four or three ISAba1 elements flanking *tra*-locus, Tn6206, and Tn6208 (Tn6209) in AbaR25 (AbaR26), respectively.

AbaR27 in BJAB0715 is a truncated version of AbaR22 with the deletion of a big portion of the sequence between *tniA* and *tetA(B)*, instead, an ISAba1 element and *sul2* gene are inserted at the same location (Fig. 5b). Unlike the AbaR25/AbaR26 which are inserted inside of *ATPase* gene in BJAB0868 and BJAB07104, the 15.3-kb AbaR27 containing *tet*A(B), *str*A, *str*B, *sul*2 resistance genes is inserted inside of a hypothetical gene similar to *EJP43116* in *A. baumannii* OIFC032 strain by using ISAba125 element in BJAB0715 (Fig. 5b). It should be noted that the novel AbaR27 is different from previously identified AbaR islands by lacking of *uspA* and *sup* genes, and not being inserted into the specific *ATPase* gene location. Also, no target site duplication was found in this resistance island in BJAB0715.

### Genes Related with MDR in Three*A. baumannii BJAB* Strains

Antimicrobial susceptibility testing showed that the 3 BJAB strains are resistant to almost all commonly used antibiotics (Table 1). The genetic variations responsible for resistance to most of the antibiotics have been identified from all BJAB strains (Table 3 and Table S2). Among the 26 drug-resistance-related genes and mutations identified from BJAB genomes, 9 of them are shared by all 3 strains and 20 are common between BJAB07104 and BJAB0868. The common drug-resistance genes shared by all 3 strains include the *str*A and *str*B (resistance to streptomycin), *tetA/B* (resistance to tetracycline), *bla_ADC_* and *bla*_OXA-23_ (resistance to carbapenems), as well as *ade* genes (*adeABC*, *adeIJK*, *adeM*) encoding for efflux pumps. A mutation (Ser83Leu) in *gyrA* gene which encodes for DNA gyrase and is responsible for resistance to fluoroquinolones was also identified in all 3 BJAB strains. BJAB07104 and BJAB0868 shared all drug resistant genes except *bla_TEM-1_* (encoding beta-lactamase class A) which is unique in BJAB0868 and is flanked by two IS26 elements [37]. BJAB0715 harbors 14 drug-resistance genes, 9 of them are shared with the other two stains and 5 are unique to BJAB0715, including *bla*_OXA58_, *aac3′-1, aphA6, cm1A1* and *bla*_OXA-10_. Interestingly, four of these unique genes in BJAB0715 are carried on its 52-kb plasmid (Fig. 2a and Table 3). Most of the drug-resistance genes are clustered on mobile genetic elements such as RI, transposons and plasmid, and therefore are transferable among different strains during the infection. As stated previously, AbaR25 and AbaR26 in BJAB07104 and BJAB0868 harbor 7 resistance genes and AbaR27 in BJAB0715 carries 5 resistance genes (*sul2, tetA(B), arsR, strB, strA*) (Fig. 5). A 20-kb plasmid identified from both BJAB0868 and BJAB07104 strains carried a group of resistance genes (*aphA1*, *sul1*, *armA*, *msrE*, *mphE*) and class I integron (*aadA1*, aac*A4* and *catB8*) (Fig. 2b). This plasmid shares 92% sequence similarity with the plasmid pZJ06 which contained all the described drug resistance genes except *catB8*. A 52-kb plasmid in BJAB0715 also carries some unique drug-resistance genes such as in *aphA6*, *bla*_OXA-58,_ *aac*3*′-I* and *cm1A1* (Fig. 2a). In addition, the drug-resistance gene clusters are always accompanied by multiple insertion elements, including ISAba1, ISAba3, IS26, ISAba125. These insertion elements may mediate the integration of resistant islands into chromosome and therefore, facilitate the transfer of drug-resistance genes among strains. On the other hand, IS elements may also enhance drug-resistance activity by promoting drug resistance gene expression [7], [38].

**Table 3:** Genes associated with Antimicrobial resistance in BJAB07104, BJAB0868 and BJAB0715.

Genes	Products	Drug-resistant function	Protein Locus Tag on BJAB genome		
			BJAB07104	BJAB0868	BJAB0715
aac A4	AAC (3)-I aminoglycoside acetyltransferase	Aminoglycoside-modifying enzymes	BJAB07104_p0002	BJAB0868_p0013	
aac 3′-I	Aminoglycoside N3′-acetyltransferase	Aminoglycoside-modifying enzymes			BJAB0715_p0027
aph A1-Iab	Aminoglycoside phosphotransferase	Aminoglycoside-modifying enzymes	BJAB07104_p0020	BJAB0868_p0011	
aphA6	Aminoglycoside phosphotransferase	Aminoglycoside-modifying enzymes			BJAB0715_p0002
aad A1	ANT (3″)-I aminoglycoside adenylyltransferase	Aminoglycoside-modifying enzymes	BJAB07104_p0004	BJAB0868_p0015	
adeT	RND (resistance-nodulation-division) family efflux pump	Efflux pumps	BJAB07104_01909	BJAB0868_02074	
adeIJK	RND (resistance-nodulation-division) family efflux pump	Efflux pumps	BJAB07104_03177-79	BJAB0868_03059-61	BJAB0715_03116-18
adeABC	RND (resistance-nodulation-division) family efflux pump	Efflux pumps	BJAB07104_01911-15	BJAB0868_02068-72	BJAB0715_00260-64
abeM	MATE (multidrug and toxic compound extrusion) family efflux pump	Efflux pumps	BJAB07104_00448	BJAB0868_00548	BJAB0715_00431
arm A	16S rRNA methylase	Aminoglycoside-modifying enzymes	BJAB07104_p0008	BJAB0868_p0019	
str A	Streptomycin resistance protein A	Aminoglycoside-modifying enzymes	BJAB07104_00282	BJAB0868_00382	BJAB0715_02883
Str B	Streptomycin resistance protein B	Aminoglycoside-modifying enzymes	BJAB07104_00281	BJAB0868_00381	BJAB0715_02882
tet A(B)	MFS (major facilitator superfamily) familyefflux pump	Ttetracycline resistance protein	BJAB07104_00277	BJAB0868_00377	BJAB0715_02878
TEM-1	Beta-lactamase class A	β-lactamases		BJAB0868_01360	
ADC	Beta-lactamase class C	β-lactamases	BJAB07104_02829	BJAB0868_02710	BJAB0715_02760
blaOXA-23	Beta-lactamase class D	β-lactamases	BJAB07104_02733	BJAB0868_00355	BJAB0715_03039
blaOXA-10	Beta-lactamase class D (OXA-51like)	β-lactamases			BJAB0715_01734,
blaOXA-66	Beta-lactamase class D (OXA-51like)	β-lactamases	BJAB07104_02182 OXA-66	BJAB0868_01795OXA-66	
blaOXA-58	Beta-lactamase class D	β-lactamases			BJAB0715_p00053
cat B8	Chloramphenicol acetyltransferase	Chloramphenicol resistance	BJAB07104_p0003	BJAB0868_p0014	
cm1A1	Chloramphenicol resistance protein	Chloramphenicol resistance			BJAB0715_p00013
mph (E)	macrolide 2′-phosphotransferase	Macrolide resistance	BJAB07104_p0012	BJAB0868_p0023	
msr E	macrolide efflux protein	Macrolide resistance	BJAB07104_p0011	BJAB0868_p0022	
sul 1	Dihydropteroate synthase	Sulphonamides	BJAB07104_p0005	BJAB0868_p0016	
gyr A a	DNA gyrase subunit A	Fluoroquinolones	BJAB07104_03067, R	BJAB0868_02946, R	BJAB0715_02991, R
par C^ b^	Topoisomerase IV subunit A		BJAB07104_00229, S	BJAB0868_00235, R	BJAB0715_00241, S

a R: Ser-Leu mutation at 83, S: no mutation at 83; ^b^: R: Ser-Leu mutation at 84, S: no mutation at 84.

An important group of drug-resistance genes identified from BJAB strains are the genes related to efflux pump function, including resistance-nodulation-cell division (RND) family, major facilitator superfamily (MFS) and multidrug and toxic efflux (MATE) family (Table S2). All three strains carry *adeABC, adeIJK*, and *abeM* genes which are important efflux pumps for multiple drug resistance in *A. baumannii* [39]–[41]. Sequence comparison revealed that these efflux genes (*adeABC, adeIJK*, and *abeM*) were conserved with almost 100% sequence similarity in all 13 *A. baumannii* strains with the exception of *adeABC* in BJAB0715 which showed 90% amino acid sequence similarity to that of *A. lwoffii* and inserted into *ATPase* (*comM* gene) position. The *adeABC* efflux pump belongs to a member of the resistance-nodulation-cell division family and can pump out multiple antibiotics and the overexpression of *adeABC* efflux pump may confer high-level resistance to carbapenems. A mechanism that controls the expression of this pump was elucidated as a two-step regulator (*adeR*) and sensor (*adeS*) system [39]. The *adeABC* efflux pump together with its regulatory proteins *adeR* and *adeS* are present in all BJAB strains, however, the mutations in *adeR* and *adeS* genes which were reported to be associated with MDR phenotype in other *A. baumannii* strains were not detected in the three BJAB strains.

Another efflux pump system identified from the BJAB strains is *tetA(B)* which drives the efflux of tetracycline (Fig. 5). The upstream of *tetA(B)* is the regulation gene *tetR*. The *tetR-tetA(B)* operon is located in the AbaR-like islands (AbaR25/AbaR26/AbaR27), same as that in other MDR *A. baumannii* strains (such as MDR-ZJ06).

*gyrA* and *parC* are intrinsic genes and point mutations in these genes confer resistance to fluoroquinolones [42], [43]. The Ser83Leu mutation in *gyrA* was detected in all three BJAB strains, but Ser84Leu mutation in *parC* was only detected in BJAB0868.

### Genes Related to Pathogenesis in BJAB Strains

O-glycosylation plays an important role in bacterial pathogenesis such as adhesion, motility, DNA uptake, protein stability, immune evasion, and animal colonization and has been reported in *A. baumannii* ATCC 17978 and other clinical isolates [44]. In BJAB strains, the presence of a general O-glycosylation system including seven glycoproteins genes as well as other pathogenesis genes related with pilus formation, hemin utilization, iron metabolism, biofilm formation, capsule formation and some putative virulence factors were verified (Table S2). Besides, the genes of phospholipase D and penicillin-binding protein 7/8 which promote the proliferation of bacteria in blood and resistance to bactericidal activity [45], [46], and outer membrane protein *ompA* which induces cytotoxicity [47] were also identified in all three BJAB strains (Table S2). Most of the pathogenesis-related genes are the same as their orthologous genes in other *A. baumannii* isolates, with the exceptions of *fimA* which is silent in BJAB0715 due to a G to A mutation.

In addition, the virulence genes encoding type IV secretary pathway such as *virB4* and *virD4* are only present in BJAB0868 and BJAB07104 with high sequence similarity to the corresponding sequence from pABTJ1 [31], but not in BJAB0715 (Table S2). It is reported that *VirB4* and *VirD4* are required at an early stage of the bacterial infection and these T4SS-associated virulence genes could be important virulence factors [48], [49]. Our data further confirmed that the *virB4/virD4* T4SS secretion system is prevalent in the epidemic *A. baumannii* clones in China. The type IV secretion system conjugation *TrbI* family proteins which were reported in AYE, ACICU and AB0057 were not found in the three BJAB strains. Furthermore, the CRISPR (clustered regularly interspaced short palindromic repeats) repeat elements, which were identified in the genomes of three GC I strains (AYE, AB0057 and AB307-0294) with a function to degrade exogenous DNA by *Cas* (CRISPR-associated) proteins [50], were not present in the BJAB strains by CRISPRFinder [51].

Furthermore, the pathogenesis islands (PIs) were predicted by PIPs software in three BJAB strains with length of 6 kb to 79 kb (Table S3). Six PIs were identified in BJAB07104, seven in BJAB0715, and four in BJAB0868. Most of the PIs are related to cell wall biogenesis, fatty acid or amino acid metabolism, drug resistance, and transport system.

### Insertion Sequence (IS) in BJAB Strains

Genome analysis of published MDR strains had identified more than 10 IS elements, including ISAba1, ISAba125, ISAba2 and IS26, but very few IS elements were found in susceptible strains. Most of the reported IS elements were also found in the genome of BJAB strains. For example, there are 14 ISAba1 and 8 ISAba125 in BJAB0715, 13 ISAba1 and 4 IS26 in BJAB0868, and 17 ISAba1 and 2 IS26 in BJAB07104. These IS elements might mediate the insertion of genetic elements into certain positions in the genome and therefore play an important role for the transition of drug resistance genes among strains. Furthermore, it has been reported that ISAba1 has promoter activity and can enhance the gene expression when located at the upstream of a gene [52]. Indeed, the ISAba1 elements were identified in the upstream of *bla_OXA-23_* and other RIs in all 3 BJAB strains, which could increase the expression of the downstream drug resistance genes. Besides, an ISAba1 element was found at the upstream of *bla_ADC_* in both BJAB0868 and BJAB07104, which can enhance the resistance to cephalosporins by potentially upregulating the expression of *bla_ADC_* in *A. baumannii* [53]. However, no IS element was identified at the upstream of *bla_ADC_* in BJAB0715, which may explain why the resistant levels to cephalosporin [ceftazidime (CAZ) and cefotaxime (CTX)] are higher in BJAB0868 and BJAB07104 (both MICs>128 µg/ml) than that in BJAB0715 (MIC 16 and 64 µg/ml for CAZ and CTX respectively).

### Genomic Variants in Three BJAB Strains

Although all three BJAB strains share high similarity in their genome, through comparative genomics analysis, we identified many genomic variants in three BJAB strains, with the scales from large structural genome re-arrangements to single nucleotide polymorphism (SNP).

**Figure 6:**
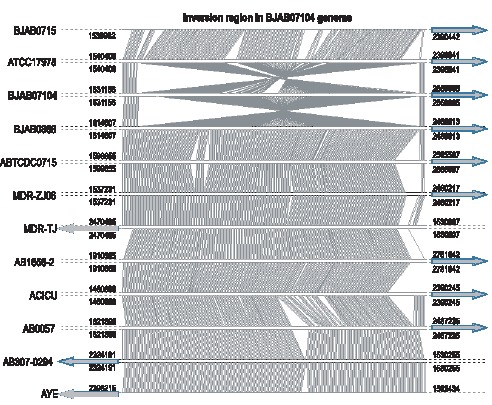
The alignment of a 800-kb inversion region in BJAB07104 genome with the genomes of other 11*A. baumannii* strains.

Genome comparison among 12 *A. baumannii* strains identified a large inverted fragment in the genome of BJAB07104 (Fig. 6) which also was verified by PCR amplification and Sanger sequencing (Fig. S3). This 800-kb inversion contains multiple transporter-related proteins. In the scope of our knowledge, this is the first report of large genomic inversion region in *A. baumannii* genome and it may represent an evolution event of clinical isolates.

By comparing genome of BJAB0715 with other whole-genome sequenced *A. baumannii* strains, we found a 10-Kb region in BJAB0715 which shares high similarity with genomic regions of three GC I strains: AB0057, AB307-0294, and AYE, but has no similarity with genomes of any GC II strains. 7 of 12 genes in this 10 kb BJAB0715 genomic region share very high protein sequence similarity in all three GC I strains (93%∼100%). For the rest 5 genes, 3 have high similarity with proteins in two of the three GC I strains, and the rest 2 genes are unique in BJAB0715 (Fig. 3b). This genome re-arrangement points out that genomic DNA transferring among different strains may not be limited by GC groups.

Genomic islands (GIs) are the most important element for acquiring foreign genes by horizontal gene transfer (HGT) [54]. We identified 16, 21 and 16 GIs by screening the genomes of BJAB07104, BJAB0715 and BJAB0868, respectively (Table S4). BJAB0868 and BJAB07104 share all of the common GIs except *bla_TEM-1_* which is absent in BJAB07104. Most of GIs identified from these two strains are also present in most of the other reported MDR strains (Table S4), suggesting that most of the prevalent MDR strains (in GC I or GC II groups) are from the same epidemic lineage. However, BJAB0715 harbors not only more number of GIs on its genome (21 vs. 16), but also contains some unique GIs which are not present on the genomes of other drug-resistant *A. baumannii* strains (Table 4 and Table S4). The existing of multiple GIs in the BJAB genome explains the rapid spreading of drug-resistance under antimicrobial selection by HGT.

**Table 4:** Major Resistant Islands (RI) in three*A. baumannii* strains and unique genomic islands (GI) in BJAB0715.

GI	BJAB0715		BJAB0868		BJAB07104		Length(bp)	G+C content	Function
	Start	End	Start	End	Start	End			
GI-715-1	275447	294778	/	/	/	/	19332	35.47	*Acinetobacter lwoffii* adeABC
GI-715-2	630027	657071	/	/	/	/	27045	35.29	Phage related
GI-715-3	2115447	2135523	/	/	/	/	20077	30.67	*Acinetobacter johnsonii* hypothetical proteins
GI-715-4	2561321	2588970	/	/	/	/	27650	39.28	Unknown
GI-715-5	3272335	3294027	/	/	/	/	21693	34.87	*Acinetobacter junii* and *A. lwoffii* hypothetical proteins
GI-715-6	3771627	3791134	/	/	/	/	19508	34.71	Unknown
GI-715-7	1061467	1075377	/	/	/	/	13911	35.79	OXA-23 island
GI-868-1	/	/	260642	381202	/	/	120561	35.68	OXA-23 island, tra system, tetA(B) island
GI-7104-1	/	/	/	/	263697	385450	121754	35.68	OXA-23 island, tra system, tetA(B) island

Another important source of genome variation which contributes to drug resistance and pathogenesis in *A. baumannii* is single nucleotide polymorphism (SNP) [55], [56]. Table S5 listed the SNPs between ACICU and the three BJAB strains using SNPFinder. BJAB07104 and BJAB0868 contain much less SNPs (10,274 and 10,766, respectively) than BJAB0715 (52,439), further indicating that BJAB0715 is much more divergent from other MDR *A. baumannii* strains. The SNP analysis result is consistent with the phylogenetic analysis using core genomes as shown in Figure 3a.

### Divergence of BJAB0715 with the Other MDR Strains

We found BJAB0715 strain shows clear divergence with other MDR strains in comparative genomics analysis. First, it is separated with other MDR strains in phylogentic analysis (Fig. 3a). Second, it is the only strain having both *bla_OXA-23_* and *bla_OXA-58_* genes. Third, BJAB0715 genome has six unique genomic islands (GI-715-1 through GI-715-6) which are not found in the genomes of other two BJAB strains (Table 4). These genomic islands (GIs) have varied sizes from 19-kb to 30-kb and they all have different G+C contents from the core genome of *A. baumannii* strains. Some of these genomic islands (GIs) shared the sequence similarity with GIs in other *Acinetobacter* species. For example, GI-715-1 contains an *adeABC* system which is similar to the one identified in *A. lwoffii* and inserted into a specific *ATPase* (*comM* gene) position which is usually an insertion hotspot for AbaR-like island in GC I and GC II clones. GI-715-3 and GI-715-5 harbor genes which have high similarity to those in *A. johnsonii* and *A junii*. GI-715-2 carries some phage-related genes. It is not clear how and why the BJAB0715 acquires various GIs from other *Acinetobacter* species. However, the divergence of BJAB0715 with other drug-resistant *A. baumannii* strains suggests that BJAB0715 is probably a newly emerged MDR strain in China.

In conclusion, in this study, we analyzed the genome of three drug resistant *A. baumannii* isolates from Beijing, China. The BJAB07104 and BJAB0868, isolated from blood and ascites samples, are genetically closest to ABTCDC0715 among whole genome sequenced *A. baumannii* strains. However, BJAB0715 is genetically more divergent to GC I and GC II strains. The identification of a 121-kb large resistance island containing transposons from several different origins and multiple drug resistance genes provided a new insight on the acquirement of drug resistance. Plasmid and insertion sequence plays an important role on HGT by direct insertion or integration into the chromosome. The evolution of *A. baumannii* clinical strains is mainly mediated by gene rearrangement such as inversion, deletion and transfer besides HGT.

## Materials and Methods

### Bacterial Isolates and Antimicrobial Susceptibility Testing (AST)

All clinical isolates of *A. baumannii* were from General Hospital of People’s Liberation Army in Beijing, China and characterized in the Clinical Microbiology Laboratory of the General Hospital of People’s Liberation Army by standard biochemical tests [20]. BJAB0715 strain was isolated from cerebrospinal fluid (CSF) sample of a patient with cerebrospinal rhinorrhea in March 2007. BJAB0868 was isolated from ascites sample of a patient with mesenteric venous thrombosis (MVT) and BJAB07104 was isolated from blood sample of a patient with liver cirrhosis in April 2008 and January 2007 respectively. All isolates were identified to the species level by the Vitek GNI system (bioMérieux, France). The MICs of several antibiotics were determined for three isolates by the agar dilution method with Müeller-Hinton agar with an inoculum of 10^4^ CFU per spot [57]. The antibiotics include imipenem (IPM), meropenem (MEM), minocycline (MNO), ciprofloxacin (CIP), levofloxacin (LVX), polymyxin, piperacillin (PIP), piperacillin/tazobactam (TZP), caftazidime, cefotaxime (CTX), cefepime (FEP) and amikacin (AMK). All protocols associated with the collection and storage of these isolates from human subjects were approved by the Hospital Review Board of the General Hospital of People’s Liberation Army. Written consent was obtained from patients for their information to be stored in the hospital database and used for research.

### Multilocus Sequence Typing (MLST)

MLST was performed based on the protocols as previously described [58]. PCR reactions were carried out in a Peltier PTC225 thermal cycler (MJ Research Inc., Watertown, MA). Sequencing reactions were performed with the ABI PRISM BigDye Terminator Cycle Sequencing Ready Reaction Kit (PerkinElmer Applied Biosystems). Data analysis and sequence alignments were carried out with the MegAlign software (DNASTAR). Sequence allele typing was performed with the multiple locus query tool at the publicly available *A. baumannii* MLST database at the Pasteur Institute’s MLST website (www.pasteur.fr/recherche/genopole/PF8/mlst/Abaumannii.html).

### Genome Sequencing and Assembling

Paired-end libraries (300–500 bp fragments) were constructed by using the Illumina ® TruSeq™ DNA Sample Preparation Kit (Illumina). Then each library was deposited onto a HiSeq Flow Cell and sequenced using an Illumina HiSeq-2000 next-generation DNA sequencer.

The Illumina short reads were assembled by VELVET to construct the contigs for each strain. Then the scaffolds and large contigs from each assembly were ordered and oriented by using the Mauve contig mover [59] and in-house script with the finished ACICU genome (GenBank accession number CP000863) as a reference. We also wrote scripts to identify un-assembled reads to fill the gaps in super-contigs and scaffolds. PCR amplification and Sanger sequencing are used to solve the ambiguity of the order and orientation of scaffolds (primer sequences and part of gel electrophoresis results were listed in Table S6 and Fig. S4–5).

### Genome Annotation

The assembled genome sequence was annotated by the NCBI Prokaryotic Genome Automatic Annotation Pipeline (PGAAP) which uses Glimmer 3.0 for identification of protein-coding genes [60], tRNAscan-SE for tRNA genes [61], and RNAmmer for rRNA genes [62]. ISs were identified using the IS Finder database (www-is.biotoul.fr) [63]. The origin of replication (oriC) and putative DnaA boxes were identified by using Ori-Finder [64]. The regions with abnormal G+C contents in the genomic sequence were obtained by using the GC-Profile program [65] to identify the genomic islands and screened in the horizontal gene transfer database (HGT-DB) [66].

### Comparative Genomics Analysis

Data used in comparative analysis were downloaded from the NCBI database (ftp://ftp.ncbi.nlm.nih.gov/GenBank/genomes/Bacteria/), including complete genome sequences and annotation of *A. baumannii* isolates MDR-ZJ06 (CP001937.1), MDR-TJ (CP003500.1), ABTCDC0715 (CP002522.1), AB1656-2 (CP001921.1), AB0057 (CP001182), AB307-0294 (CP001172), ATCC 17978 (CP000521), ACICU (CP000863), AYE (CU459141), SDF (CU468230), and ADP1 (CR543861.1). Multiple sequence alignments and comparison analysis of these genomes were performed with Mauve [67], [68] and ACT (http://www.sanger.ac.uk/Software/ACT) [69]. BLASTP was used to compare proteins from each pair of genomes to identify the best reciprocal matches with cutoff of >50% amino acid similarity and >80% coverage in length. PHYLIP package (ver. 3.69) was used to construct the phylogram of the 1,119 orthologous proteins with 1000 replicates in bootstrap. Mauve, IslandViewer and in-house-developed Perl scripts were used to identify the potential genomic islands [67], [70]. SNPFinder and Mauve were used to identify SNPs [67], [71]. PIPs [72] was used to predict the potential pathogenicity islands with SDF as a reference strain.

### Southern Blot Analysis and Location of*bla*_OXA-23_ Gene

To determine the location of the *bla*_OXA-23_ gene and *tra*-locus, chromosomal and plasmid DNA in two isolates of BJAB07104 and BJAB0868 were evaluated by Southern blot analysis. Genomic DNA was prepared with Wizard Genomic DNA Purification Kit (Promega, Madison, Wis.). Extraction of plasmid DNA was performed using the Kieser method as described previously [73]. Genomic and plasmid DNAs were digested using BamHI/BglII, separated by electrophoresis on 0.8% agarose gels, and transferred onto Hybond N+ membranes (Amersham International, Buckinghamshire, England) as described by Sambrook and Russell [74]. Labeling of probes (522-bp of *bla*_OXA-23_ amplicon generated with primers OXA-23-L and OXA-23-R, and 920-bp of *virD4* amplicon generated with primers virD4-L and virD4-R) were performed with digoxigenin as described by the manufacturer (Roche Diagnostics GmbH, Mannheim, Germany). Southern hybridization was performed at 68°C with the buffers recommended in the instructions included in the digoxigenin kit from Roche.

### Nucleotide Sequence Accession Numbers

The complete genome sequences of *Acinetobacter baumannii* strains BJAB07104, BJAB0715 and BJAB0868 and plasmids pBJAB07104, p1BJAB0868, p2BJAB0868, pBJAB0715 reported in this paper have been deposited in the GenBank (http://www.ncbi.nlm.nih.gov/genbank/) under accession numbers CP003846, CP003849, CP003847, CP003887, CP003850, CP003888, and CP003848 respectively. In addition, the sequences of AbaR25 and AbaR26 have been deposited in the GenBank under accession numbers CP003907 and CP003908 respectively.

## Supporting Information

Figure S1 **Gel electrophoresis of the sequencing assembly of Tn6206 and tra-locus in chromosome and free plasmids verified by PCR amplification in BJAB07104 (a) and in BJAB0868 (b).** All the expected PCR products were sequenced by DNA Sanger sequencing.
(PPTX)

Figure S2 **Identification of the localization of Tn6206 and tra-locus in chromosomal DNA and plasmid DNA by Southern blot.** (a) Hybridization of the BamHI/BglII-fragments with a *bla*_OXA-23_ probe. The chromosome-integrated fragment (Tn6206->*tra*) produced one band (11337 bp for BJAB07104, and 11336 bp for BJAB0868); and the chromosome-integrated fragment (*tra*->Tn6206) produced one band (7943 bp for BJAB07104 and BJAB0868); the free plasmid produced one band (7943 bp for a plasmid containing *tra*+Tn6206, and 7245 bp for a plasmid containing only Tn6206). (b) Hybridization of the BamHI/BglII-fragments with a virD4 probe. Both chromosome-integrated fragments (Tn6206->*tra*, *tra*->Tn6206) and the free plasmids (containing Tn6206+*tra*, or containing only *tra*) produced a 1418-bp fragment.
(PPTX)

Figure S3 **Gel electrophoresis of the large inversion verified by PCR amplification in BJAB07104.** All the expected PCR products were confirmed by Sanger sequencing.
(PPTX)

Figure S4 **Gel electrophoresis of gap-closing PCR in BJAB07104.** All the expected PCR products were confirmed by Sanger sequencing.
(PPTX)

Figure S5 **Gel electrophoresis of gap-closing PCR in BJAB0715.** All the expected PCR products were confirmed by Sanger sequencing.
(PPTX)

Table S1 **List of 1119 conserved genes among all 14** ***Acinetobacter baumannii*** **strains.**
(XLSX)

Table S2 **The genes associated with resistance and pathogenesis in three BJ strains.**
(XLSX)

Table S3 **The predicted pathogenicity islands in three BJAB strains.**
(XLSX)

Table S4 **The genomic islands and their functions in three BJAB strains.**
(XLSX)

Table S5 **SNPs analysis in three** ***A. baumannii*** **strains.**
(XLSX)

Table S6 **Primer sequences for gap-closing PCR.**
(XLSX)
