# Supplementary material for: Complete Genome Analysis of Three Acinetobacter baumannii Clinical Isolates in China for Insight into the Diversification of Drug Resistance Elements
Source: PLoS One. 2013 Jun 24;8(6):e66584. doi: 10.1371/journal.pone.0066584 (PMC3691203; doi:10.1371/journal.pone.0066584)
Supplement: Figure S1 — Gel electrophoresis of the sequencing assembly of Tn6206 and tra-locus in chromosome and free plasmids verified by PCR amplification in BJAB07104 (a) and in BJAB0868 (b). All the expected PCR products were sequenced by DNA Sanger sequencing. (PPTX) [file pone.0066584.s001.pptx]

## Slide 1
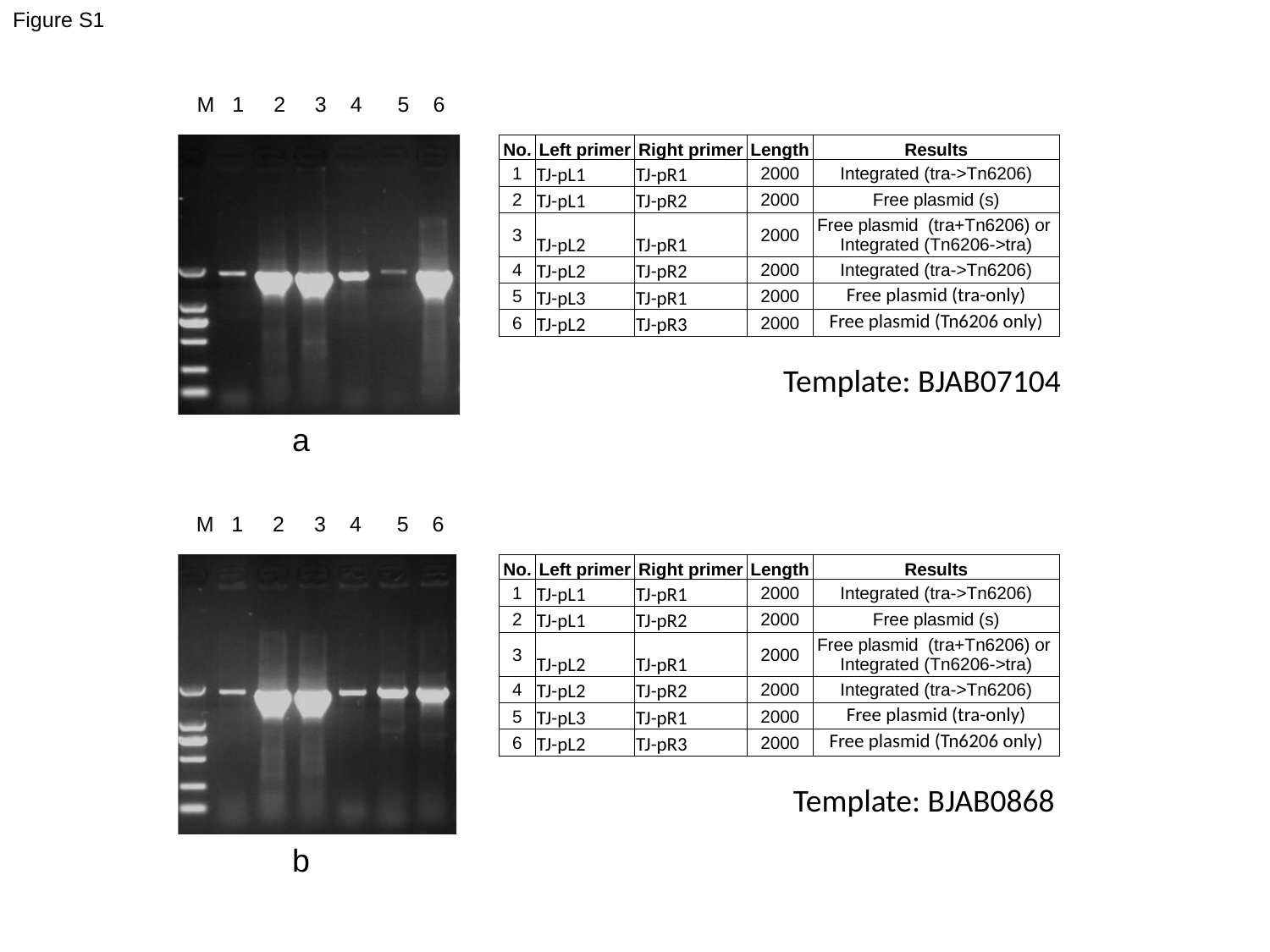

Figure S1
 M 1 2 3 4 5 6
| No. | Left primer | Right primer | Length | Results |
| --- | --- | --- | --- | --- |
| 1 | TJ-pL1 | TJ-pR1 | 2000 | Integrated (tra->Tn6206) |
| 2 | TJ-pL1 | TJ-pR2 | 2000 | Free plasmid (s) |
| 3 | TJ-pL2 | TJ-pR1 | 2000 | Free plasmid (tra+Tn6206) or Integrated (Tn6206->tra) |
| 4 | TJ-pL2 | TJ-pR2 | 2000 | Integrated (tra->Tn6206) |
| 5 | TJ-pL3 | TJ-pR1 | 2000 | Free plasmid (tra-only) |
| 6 | TJ-pL2 | TJ-pR3 | 2000 | Free plasmid (Tn6206 only) |
Template: BJAB07104
a
 M 1 2 3 4 5 6
| No. | Left primer | Right primer | Length | Results |
| --- | --- | --- | --- | --- |
| 1 | TJ-pL1 | TJ-pR1 | 2000 | Integrated (tra->Tn6206) |
| 2 | TJ-pL1 | TJ-pR2 | 2000 | Free plasmid (s) |
| 3 | TJ-pL2 | TJ-pR1 | 2000 | Free plasmid (tra+Tn6206) or Integrated (Tn6206->tra) |
| 4 | TJ-pL2 | TJ-pR2 | 2000 | Integrated (tra->Tn6206) |
| 5 | TJ-pL3 | TJ-pR1 | 2000 | Free plasmid (tra-only) |
| 6 | TJ-pL2 | TJ-pR3 | 2000 | Free plasmid (Tn6206 only) |
Template: BJAB0868
b
