# Supplementary material for: Complete Genome Analysis of Three Acinetobacter baumannii Clinical Isolates in China for Insight into the Diversification of Drug Resistance Elements
Source: PLoS One. 2013 Jun 24;8(6):e66584. doi: 10.1371/journal.pone.0066584 (PMC3691203; doi:10.1371/journal.pone.0066584)
Supplement: Figure S2 — Identification of the localization of Tn6206 and tra-locus in chromosomal DNA and plasmid DNA by Southern blot. (a) Hybridization of the BamHI/BglII-fragments with a bla OXA-23 probe. The chromosome-integrated fragment (Tn6206->tra) produced one band (11337 bp for BJAB07104, and 11336 bp for BJAB0868); and the chromosome-integrated fragment (tra->Tn6206) produced one band (7943 bp for BJAB07104 and BJAB0868); the free plasmid produced one band (7943 bp for a plasmid containing tra+Tn6206, and 7245 bp for a plasmid containing only Tn6206). (b) Hybridization of the BamHI/BglII-fragments with a virD4 probe. Both chromosome-integrated fragments (Tn6206->tra, tra->Tn6206) and the free plasmids (containing Tn6206+tra, or containing only tra) produced a 1418-bp fragment. (PPTX) [file pone.0066584.s002.pptx]

## Slide 1
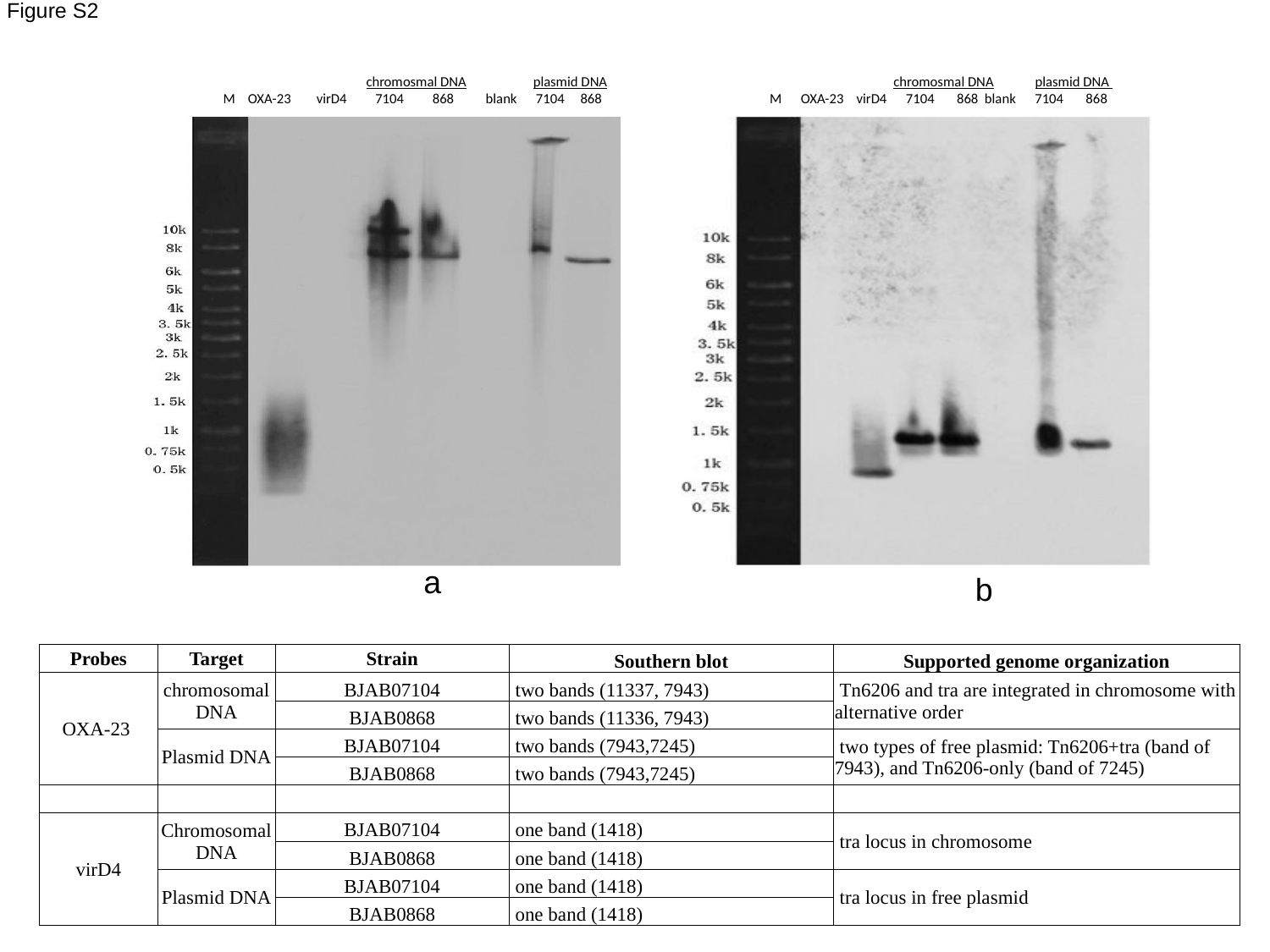

Figure S2
 chromosmal DNA plasmid DNA
 M OXA-23 virD4 7104 868 blank 7104 868
 chromosmal DNA plasmid DNA
 M OXA-23 virD4 7104 868 blank 7104 868
a
b
| Probes | Target | Strain | Southern blot | Supported genome organization |
| --- | --- | --- | --- | --- |
| OXA-23 | chromosomal DNA | BJAB07104 | two bands (11337, 7943) | Tn6206 and tra are integrated in chromosome with alternative order |
| | | BJAB0868 | two bands (11336, 7943) | |
| | Plasmid DNA | BJAB07104 | two bands (7943,7245) | two types of free plasmid: Tn6206+tra (band of 7943), and Tn6206-only (band of 7245) |
| | | BJAB0868 | two bands (7943,7245) | |
| | | | | |
| virD4 | Chromosomal DNA | BJAB07104 | one band (1418) | tra locus in chromosome |
| | | BJAB0868 | one band (1418) | |
| | Plasmid DNA | BJAB07104 | one band (1418) | tra locus in free plasmid |
| | | BJAB0868 | one band (1418) | |
