# Supplementary material for: Complete Genome Analysis of Three Acinetobacter baumannii Clinical Isolates in China for Insight into the Diversification of Drug Resistance Elements
Source: PLoS One. 2013 Jun 24;8(6):e66584. doi: 10.1371/journal.pone.0066584 (PMC3691203; doi:10.1371/journal.pone.0066584)
Supplement: Figure S3 — Gel electrophoresis of the large inversion verified by PCR amplification in BJAB07104. All the expected PCR products were confirmed by Sanger sequencing. (PPTX) [file pone.0066584.s003.pptx]

## Slide 1
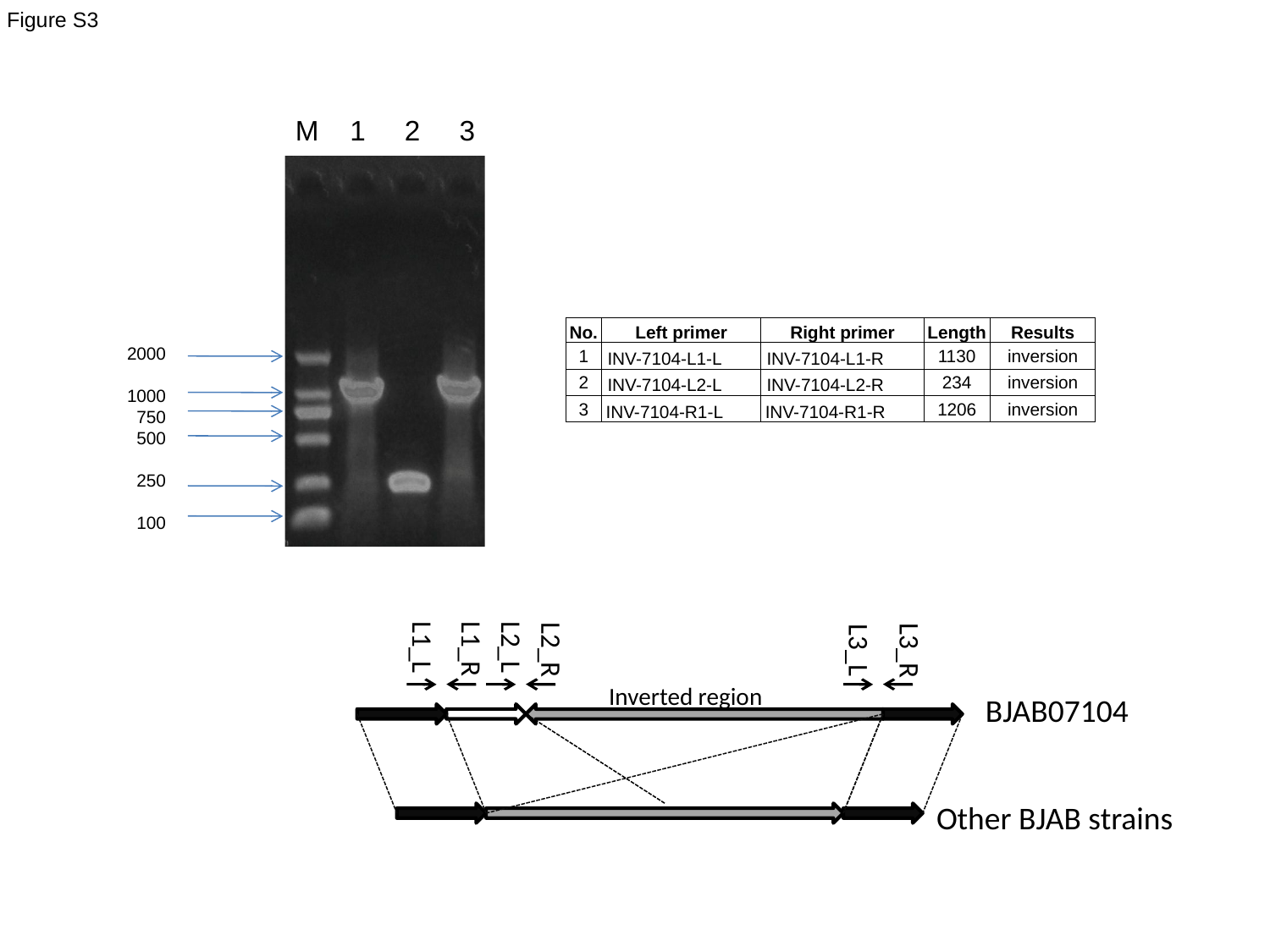

Figure S3
 M 1 2 3
| No. | Left primer | Right primer | Length | Results |
| --- | --- | --- | --- | --- |
| 1 | INV-7104-L1-L | INV-7104-L1-R | 1130 | inversion |
| 2 | INV-7104-L2-L | INV-7104-L2-R | 234 | inversion |
| 3 | INV-7104-R1-L | INV-7104-R1-R | 1206 | inversion |
2000
 1000
750
500
250
100
L1_L
L1_R
L2_L
L2_R
L3_R
L3_L
Inverted region
BJAB07104
Other BJAB strains
