# Supplementary material for: Complete Genome Analysis of Three Acinetobacter baumannii Clinical Isolates in China for Insight into the Diversification of Drug Resistance Elements
Source: PLoS One. 2013 Jun 24;8(6):e66584. doi: 10.1371/journal.pone.0066584 (PMC3691203; doi:10.1371/journal.pone.0066584)
Supplement: Figure S4 — Gel electrophoresis of gap-closing PCR in BJAB07104. All the expected PCR products were confirmed by Sanger sequencing. (PPTX) [file pone.0066584.s004.pptx]

## Slide 1
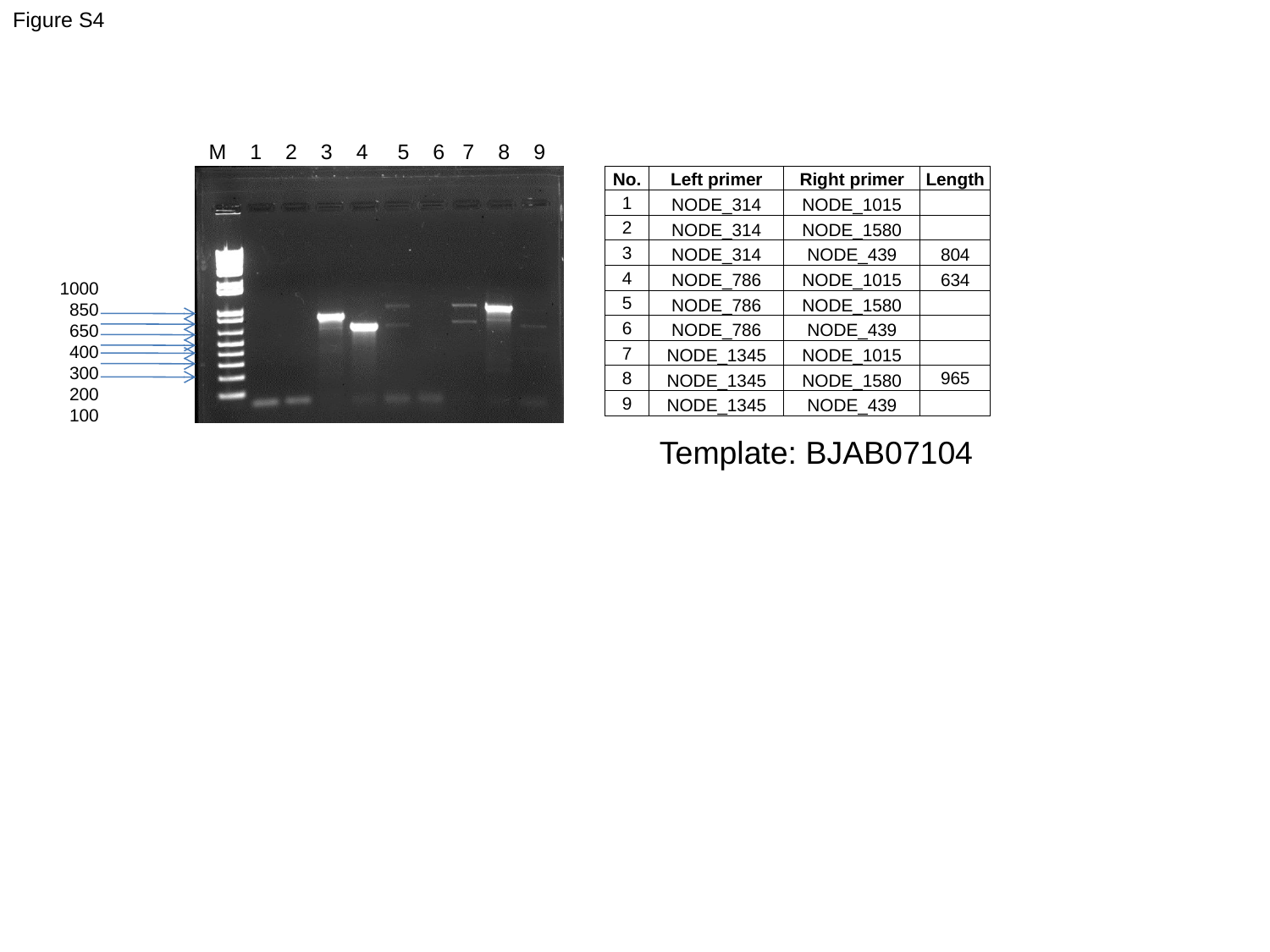

Figure S4
M 1 2 3 4 5 6 7 8 9
| No. | Left primer | Right primer | Length |
| --- | --- | --- | --- |
| 1 | NODE\_314 | NODE\_1015 | |
| 2 | NODE\_314 | NODE\_1580 | |
| 3 | NODE\_314 | NODE\_439 | 804 |
| 4 | NODE\_786 | NODE\_1015 | 634 |
| 5 | NODE\_786 | NODE\_1580 | |
| 6 | NODE\_786 | NODE\_439 | |
| 7 | NODE\_1345 | NODE\_1015 | |
| 8 | NODE\_1345 | NODE\_1580 | 965 |
| 9 | NODE\_1345 | NODE\_439 | |
1000
850
650
400
300
200
100
Template: BJAB07104
