# Supplementary material for: Complete Genome Analysis of Three Acinetobacter baumannii Clinical Isolates in China for Insight into the Diversification of Drug Resistance Elements
Source: PLoS One. 2013 Jun 24;8(6):e66584. doi: 10.1371/journal.pone.0066584 (PMC3691203; doi:10.1371/journal.pone.0066584)
Supplement: Figure S5 — Gel electrophoresis of gap-closing PCR in BJAB0715. All the expected PCR products were confirmed by Sanger sequencing. (PPTX) [file pone.0066584.s005.pptx]

## Slide 1
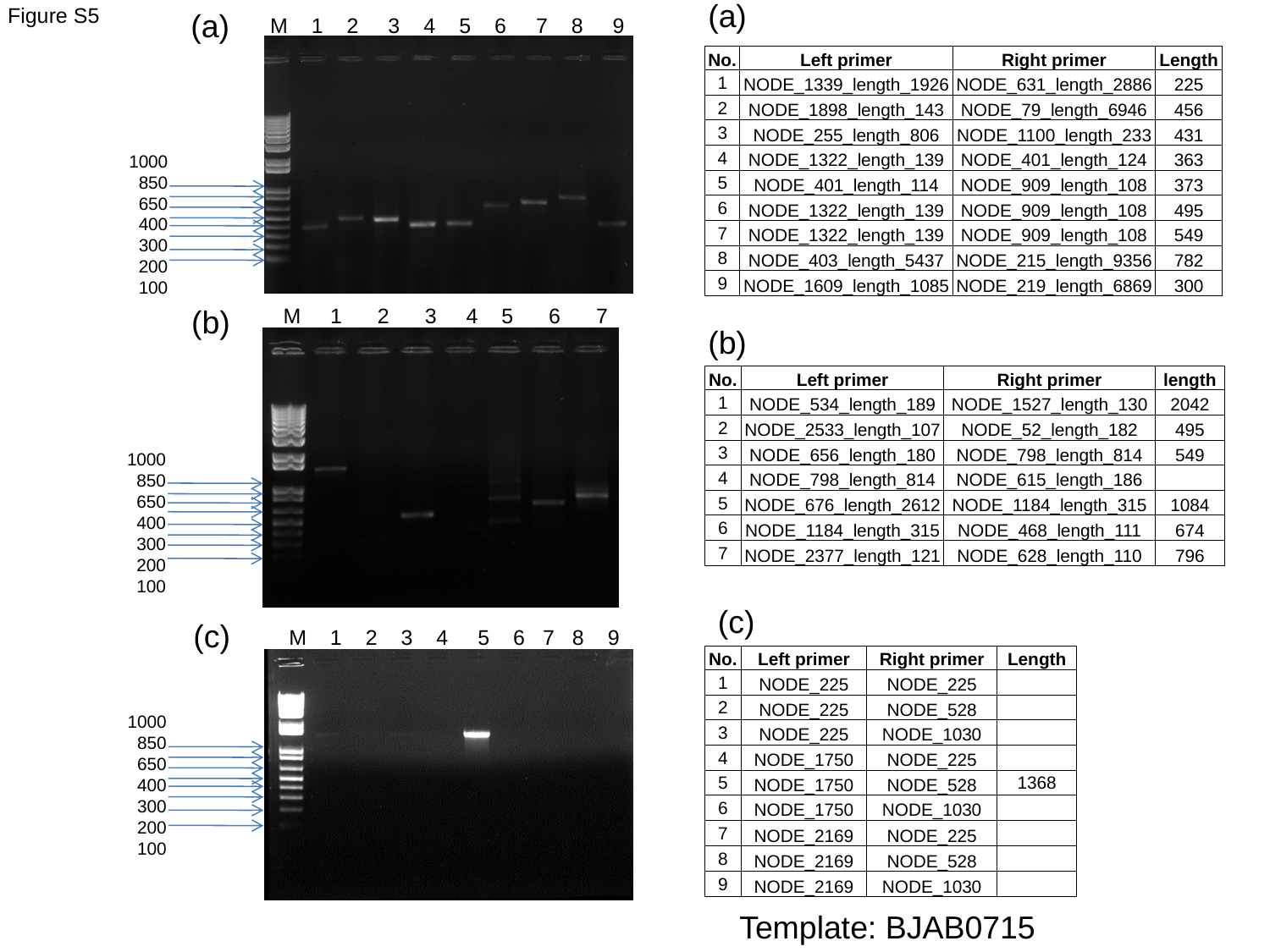

(a)
(a)
Figure S5
M 1 2 3 4 5 6 7 8 9
| No. | Left primer | Right primer | Length |
| --- | --- | --- | --- |
| 1 | NODE\_1339\_length\_1926 | NODE\_631\_length\_2886 | 225 |
| 2 | NODE\_1898\_length\_143 | NODE\_79\_length\_6946 | 456 |
| 3 | NODE\_255\_length\_806 | NODE\_1100\_length\_233 | 431 |
| 4 | NODE\_1322\_length\_139 | NODE\_401\_length\_124 | 363 |
| 5 | NODE\_401\_length\_114 | NODE\_909\_length\_108 | 373 |
| 6 | NODE\_1322\_length\_139 | NODE\_909\_length\_108 | 495 |
| 7 | NODE\_1322\_length\_139 | NODE\_909\_length\_108 | 549 |
| 8 | NODE\_403\_length\_5437 | NODE\_215\_length\_9356 | 782 |
| 9 | NODE\_1609\_length\_1085 | NODE\_219\_length\_6869 | 300 |
1000
850
650
400
300
200
100
(b)
M 1 2 3 4 5 6 7
(b)
| No. | Left primer | Right primer | length |
| --- | --- | --- | --- |
| 1 | NODE\_534\_length\_189 | NODE\_1527\_length\_130 | 2042 |
| 2 | NODE\_2533\_length\_107 | NODE\_52\_length\_182 | 495 |
| 3 | NODE\_656\_length\_180 | NODE\_798\_length\_814 | 549 |
| 4 | NODE\_798\_length\_814 | NODE\_615\_length\_186 | |
| 5 | NODE\_676\_length\_2612 | NODE\_1184\_length\_315 | 1084 |
| 6 | NODE\_1184\_length\_315 | NODE\_468\_length\_111 | 674 |
| 7 | NODE\_2377\_length\_121 | NODE\_628\_length\_110 | 796 |
1000
850
650
400
300
200
100
(c)
(c)
M 1 2 3 4 5 6 7 8 9
| No. | Left primer | Right primer | Length |
| --- | --- | --- | --- |
| 1 | NODE\_225 | NODE\_225 | |
| 2 | NODE\_225 | NODE\_528 | |
| 3 | NODE\_225 | NODE\_1030 | |
| 4 | NODE\_1750 | NODE\_225 | |
| 5 | NODE\_1750 | NODE\_528 | 1368 |
| 6 | NODE\_1750 | NODE\_1030 | |
| 7 | NODE\_2169 | NODE\_225 | |
| 8 | NODE\_2169 | NODE\_528 | |
| 9 | NODE\_2169 | NODE\_1030 | |
1000
850
650
400
300
200
100
Template: BJAB0715
